# Protlego: a Python package for the analysis and design of chimeric proteins

**DOI:** 10.1093/bioinformatics/btab253

**Published:** 2021-04-26

**Authors:** Noelia Ferruz, Jakob Noske, Birte Höcker

**Affiliations:** Department of Biochemistry, University of Bayreuth, 95447 Bayreuth, Germany; Department of Biochemistry, University of Bayreuth, 95447 Bayreuth, Germany; Department of Biochemistry, University of Bayreuth, 95447 Bayreuth, Germany

## Abstract

**Motivation:**

Duplication and recombination of protein fragments have led to the highly diverse protein space that we observe today. By mimicking this natural process, the design of protein chimeras via fragment recombination has proven experimentally successful and has opened a new era for the design of customizable proteins. The *in silico* building of structural models for these chimeric proteins, however, remains a manual task that requires a considerable degree of expertise and is not amenable for high-throughput studies. Energetic and structural analysis of the designed proteins often require the use of several tools, each with their unique technical difficulties and available in different programming languages or web servers.

**Results:**

We implemented a Python package that enables automated, high-throughput design of chimeras and their structural analysis. First, it fetches evolutionarily conserved fragments from a built-in database (also available at fuzzle.uni-bayreuth.de). These relationships can then be represented via networks or further selected for chimera construction via recombination. Designed chimeras or natural proteins are then scored and minimized with the Charmm and Amber forcefields and their diverse structural features can be analyzed at ease. Here, we showcase Protlego’s pipeline by exploring the relationships between the P-loop and Rossmann superfolds, building and characterizing their offspring chimeras. We believe that Protlego provides a powerful new tool for the protein design community.

**Availability and implementation:**

Protlego runs on the Linux platform and is freely available at (https://hoecker-lab.github.io/protlego/) with tutorials and documentation.

**Supplementary information:**

Supplementary data are available at *Bioinformatics* online.

## 1 Introduction

Proteins evolved to form diverse structures and perform a multitude of functions. If we could unravel basic rules to design new proteins and implement tailored functions, this would address many challenges of today’s society. The design of customized proteins, however, has not been an easy task. There are by now impressive examples for *de novo* designed protein structures (Huang *et al*., 2014, 2016; Kuhlman *et al*., 2003; Thomson *et al*., 2014). Yet, the majority of engineered enzymes are still obtained via directed evolution starting from a natural protein (Lechner et al., 2018). In addition, the recombination or duplication of natural protein segments has led to new proteins (Höcker, 2014). Nature seems to have created the vast protein space by these latter mechanisms, i.e. via the duplication and recombination of protein parts. Domain recombination has led to the development of large multidomain proteins, whose synergic effects enable differentiation and speciation of functionalities (Dohmen *et al*., 2020). Domains are allegedly the basic evolutionary unit (Apic and Russell, 2010; Ponting and Russell, 2002), and significant efforts have been made to hierarchically classify them, such as in the SCOP (Fox *et al*., 2014), CATH (Dawson *et al*., 2017) and ECOD (Cheng *et al*., 2014) databases. Moreover, the origin of domains themselves is known today to derive from the duplication, recombination and differentiation of sub-domain sized fragments (Lupas *et al*., 2001; Söding and Lupas, 2003).

For the TIM-barrel and the flavodoxin-like fold, we could show that these two major protein folds are evolutionarily related and share a fragment of common origin (Farías-Rico *et al*., 2014). Further, Alva *et al.* identified a set of 40 peptides of up to 38 amino acids in length whose sequence similarity is evidence of common ancestry despite appearing in different folds (Alva *et al*., 2015). Similarly, we performed an all-against-all comparison of protein domains representing all existing folds and identified more than 1000 conserved protein fragments of various lengths across protein space. These fragments represent building blocks that nature has reused throughout evolution and that can now be browsed in the Fuzzle database (fuzzle.uni-bayreuth.de) (Ferruz *et al*., 2020).

Engineering efforts have been successful in designing new protein domains by duplicating or recombining protein fragments. The design of symmetric protein structures through duplication of the same fragment has been achieved for several folds (Fortenberry *et al*., 2011; Höcker *et al*., 2004; Reichen *et al*., 2016; Yadid and Tawfik, 2011). The design via recombination of fragments within as well as across folds has also been effective (Höcker, 2014). The TIM-barrel protein HisF and the flavodoxin-like domain CheY were combined and optimized yielding a robust and well-folded chimera (Bharat *et al*., 2008; Eisenbeis *et al.*, 2012). In a follow-up study, the combination of HisF with NarL led to a protein of even higher stability (Shanmugaratnam *et al*., 2012).

While design via recombination is fairly new, its success provides an alternative to classical approaches. Individual fragments can contribute their unique functional properties to the chimeric protein, which provides an interesting, and generalizable route for protein design (Höcker, 2014). However, the *in silico* automated design of chimeric proteins as a prior step for experimental studies requires broad expertise and the use of several tools (Farias-Rico and Höcker, 2013).

A few algorithms have been made available for the recombination of sequences. SCHEMA detects segments of homologous proteins that can be recombined without disturbing the integrity of the structures. The resulting sequences produced folded proteins with a greater likelihood than by random shuffling (Meyer *et al*., 2003; Voigt *et al*., 2002). Later the RASPP algorithm was described, which creates chimera libraries enriched in folded proteins without compromising the diversity (RASPP) (Endelman *et al*., 2004). In addition, the software MODELLER is a useful tool for modelling of protein structures based on a sequence alignment (Sánchez and Sali, 2000), and thus has been used for some chimeras (Farias-Rico and Höcker, 2013). More recently, machine learning methods have led to higher-accuracy predictors, such as AlphaFold (Senior *et al*., 2020) or DMPfold (Greener *et al*., 2019). The Kuhlman lab implemented SEWING, a computational approach which enabled the creation of chimeric all-alpha structures by joining short αhelical fragments (Jacobs *et al*., 2016) and optimizing the chimeras with the program ROSETTA (Leaver-Fay *et al*., 2011). To our knowledge, the method has not been applied to the construction of other than all-α chimeras.

Here, we present an easy-to-use python package named Protlego that automates the process of *in silico* chimera design and structural analysis. We showcase Protlego’s features by exploring the relationships between two α/βsuperfolds, namely, the P-loop containing nucleoside triphosphate hydrolases (NTPases) and the Rossmann fold. Tutorials to reproduce these results and guidelines to customize analysis are available at https://hoecker-lab.github.io/protlego/ and Supplementary Listing S1. We believe Protlego will be useful for protein engineers and evolutionary biologists alike.

## 2 Materials and methods

### 2.1 Fetching hits from the Fuzzle database

**Fig. 1.**
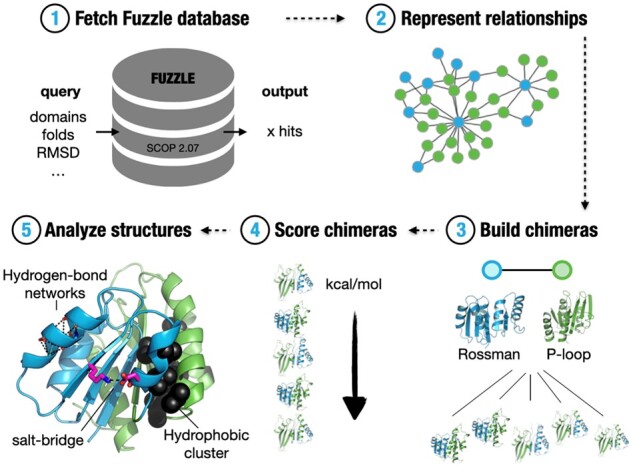
Overview of Protlego features. Protlego is mainly divided into five applications: It is possible to fetch from a built-in Fuzzle database (1) and represent the hits via similarity networks (2). The hits can also be used to build chimeras (3), which can later be scored (4) and analyzed (5)

Protlego contains a lightweight Fuzzle database that gets installed during setup via sqlite3. The database was created by an all-against-all profile hidden Markov model (HMM) comparison of all domains in SCOPe 2.07 (Fox *et al.*, 2014) using HHsearch (Söding, 2005). Fuzzle contains more than 10 million hits among over 28 000 unique domains (Fig. 1). Each hit in Fuzzle contains information about the two domains that contain a common fragment (denoted *query* and *subject*), start and end of the fragment they share, HHsearch probability and RMSD, among others (Ferruz *et al.*, 2020). Protlego enables fetching hits from Fuzzle searching via PDB identifier (Berman, 2000), domain identifier, or specific SCOPe group (families, superfamilies and folds). It is also possible to fetch entire subspaces that fulfil certain criteria (e.g. RMSD below a certain threshold). An overview of the methods available in this application is shown in Supplementary Table S1.

### 2.2 Network visualization

Protlego facilitates the visualization of evolutionary relationships via similarity networks (Fig. 1). The Graph class takes a hit or a set of hits from the built-in database and enables its representation harnessing the power of the *graph-tool* package (https://graph-tool.skewed.de/) . Network nodes represent protein domains and links join domains that have a fragment in common. It is possible to directly visualize nodes (the fragment in the context of its domain) and links (the alignments between two domains) via Protlego’s VMD integration (Humphrey *et al*., 1996).

### 2.3 Chimera modelling

**Fig. 2.**
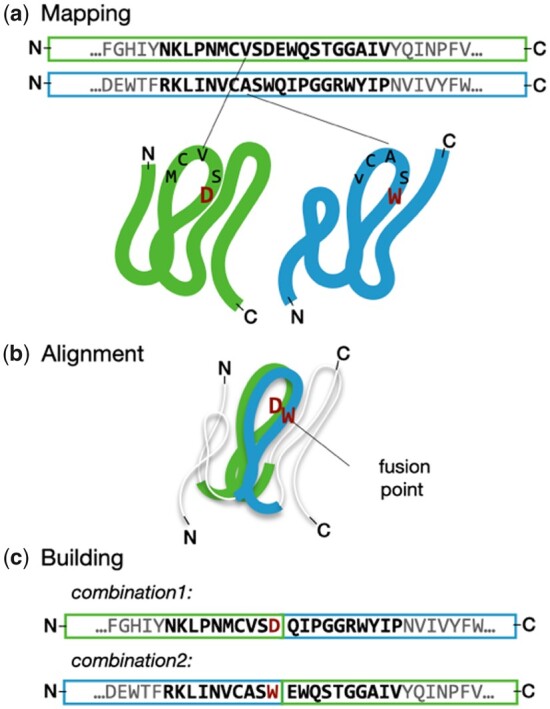
Chimera building in Protlego. The amino acids in the HHsearch alignment are mapped onto the corresponding PDB structures (**a**). These fragments are then superimposed by minimizing the RMSD of each pair of alpha carbons with TMalign (**b**). Distances for each fusion are computed and each one below a cut-off is defined as a fusion point. Each fusion point can produce 2 chimeras, one with the query N-terminus (combination1) and the other with the subject N-terminus (combination2) (**c**). The process is repeated for each fusion point. Chimeras are accepted if no backbone-backbone clashes are observed

Protlego builds all possible chimeras between two protein parents (Figs 1 and 2). The chimeras are built by combining N- and C-terminus from query and subject, and thus present a single recombination point. All possible chimeras from the two combinations are built, where *combination1* refers to those chimeras where the N-terminus comes from the query, and *combination2* to those where it comes from the subject. The Builder class takes a hit as an argument and uses the HHsearch alignment as a template to create the models. The amino acids in the local alignment get mapped to their corresponding alpha carbon atoms in the two PDB structures (Fig. 2a). The alignment of these PDB structures is performed with TMalign (Zhang and Skolnick, 2005) taking only into account the fragment’s Cα atoms. There are two ways to perform this alignment, either by minimizing the RMSD taking all the alpha carbons into account in a global fashion, or by performing stepwise partial alignments. In the case of a long fragment, the partial mode iteratively finds the best alignments for shorter regions such as, for example, βα-motifs. These regions are defined by the sequence alignment: sequence alignment gaps constitute the boundaries that define each shorter region. Once the alignment is performed, Protlego computes the distance of each pair of aligned alpha carbons (Fig. 2b).

Low distances, sometimes found in secondary structure elements, confer ideal fusion points for recombination, as the merging of the two parents ensures that the resulting structure will be minimally perturbed. We use the default value of 1 Å as a maximum distance, albeit users can define their own thresholds. Once the optimal fusion points are found, we recombine query and subjects at these positions (Fig. 2c). Only chimeras that do not present backbone-backbone clashes are kept. The handling of PDBs is performed with the *moleculekit* package (Doerr *et al*., 2016).

### 2.4 Potential energy evaluation

Protlego allows the estimation of the potential energy of Chimera objects with the Charmm (Vanommeslaeghe *et al*., 2009) and Amber (Ponder and Case, 2003) forcefields (Fig. 1). The structures can be scored or minimized accounting the protein backbone when desired. GPU acceleration is supported. This potential energy estimation uses the functionality of the *openMM* package (Eastman *et al*., 2017).

### 2.5 Structural analysis

Protlego enables the computation of several properties for the designed chimeras or natural proteins (Fig. 1). The user can automatically fetch a PDB or SCOPe domain within the Chimera class. Among others, the computation of solvent accessible surface area (SASA), distance matrices, contact orders, contact maps and *HHbond* plots (Bikadi *et al*., 2007) is implemented. Protlego’s VMD integration (Humphrey *et al.*, 1996) enables the automatic visualization of structural features in the protein. The computation of hydrogen networks utilizes some of the functionality of the *MDtraj* package (McGibbon *et al*., 2015). Protlego also includes a Python reimplementation of the CSU algorithm (Sobolev *et al*., 1999) which enables the computation of hydrophobic clusters in a high-throughput fashion (Supplementary Text S1).

## 3 Results

**Fig. 3.**
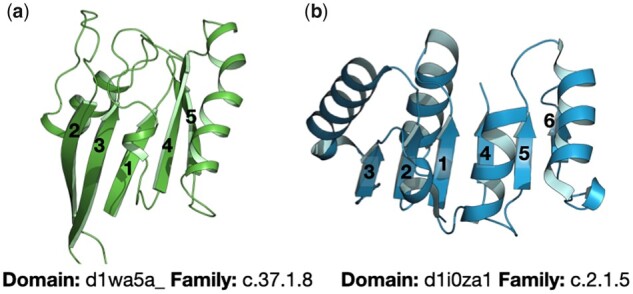
Exemplary topologies of the P-loop (**a**) and Rossman folds (**b**). Both folds belong to the α/β class. Whereas the P-loop containing nucleoside triphosphate hydrolases (SCOPe classification: c.37) have a 5- or 6-stranded β-sheet in the order 23145(6), the NAD(P)-binding Rossmann fold (c.2) has a 6-stranded β-sheet with the order 321456

Here, we present Protlego’s main applications by showcasing the example of the P-loop and Rossman α/β-superfolds. The P-loop NTPases are a superfamily of enzymes that catalyze the hydrolysis of nucleoside triphosphate molecules (NTP). Despite extreme sequence and topology divergence, P-loop proteins are characterized by the presence of the sequence pattern GxxxxGKS/T known as the Walker A motif (Walker *et al*., 1982), which binds the terminal phosphate groups of NTPs, and the flanking β-strand and α-helix (Romero Romero *et al*., 2018). Due to their sequence dissimilarity, several attempts have been made to classify P-loop proteins (Leipe *et al*., 2003; Lupas and Martin, 2002; Pathak *et al*., 2014). SCOPe classifies P-loop NTPases within the c.37 fold, all belonging to the c.37.1 superfamily (P-loop containing nucleoside triphosphate hydrolases). The domains are divided into 26 families based on their β-sheet topologies. While all P-loop NTPases have three layers, with two and three helices sandwiching a 5- or 6-stranded parallel β-sheet, the order of strands varies. The great majority has the order 23145(6) as shown in Figure 3a.

The Rossmann fold is another of the most ancient and functionally diverse folds, catalyzing more than 300 enzymatic reactions (Bukhari and Caetano-Anollés, 2013). Like the P-loop fold, most Rossmann enzymes use NTPs as cofactors and are formed by an α/β-sandwich, in this case by 6 parallel β-strands with the order 321456 (Fig. 3b). Similar to P-loop NTPases, Nicotinamide adenine dinucleotide (NAD) and flavin adenine dinucleotide (FAD)-utilizing enzymes contain a Gly-rich motif that resides between α1 and β1 and is able to bind the phosphate group of several NTPs. Besides, Rossman domains usually have an Asp/Glu residue at the top of β2 that provides a conserved and well-studied carboxylate-ribose bidentate interaction (Laurino *et al*., 2016).

The two folds comprise highly similar topologies but deviate by the order of the core β-strands (23145 versus 321456) and their different binding motifs at their N-termini. We explored the interaction between these α/β-superfolds further to (i) determine whether there are homologous regions in their sequence and (ii) showcase the application of the Protlego package on an interesting example. The code and computational costs to reproduce these examples is provided in Supplementary Listing S1.

### 3.1 Fetching related fragments from the P-loop and Rossmann folds

We first fetched all hits between the P-loop and Rossmann folds in the built-in Fuzzle database. The corresponding SCOPe identifiers for these folds are c.37 and c.2, respectively. We filtered for hits that have an HHsearch probability > 70%, an RMSD < 3 Å, fragment length between 10 and 200 amino acids, and TM-score below 0.3 in line with previous studies (Alva *et al.*, 2015). The fetch_group function retrieved 1737 hits, containing 432 different unique domains. Each hit contains information about query and subject, start and end of the fragment they share, HHsearch probability, and RMSD, among others. The hits had an average length of 37.9 ± 17.0 amino acids (median 34.0) with a bimodal distribution with centres at 38 and 105 amino acids (Supplementary Fig. S2), but they markedly lean towards short length: 93% of the hits are below 45 amino acids, and only 5% over 100. Regarding other average properties, the hits have a mean RMSD of 2.3 ± 0.3 Å, an HHsearch probability of 77.0 ± 5% and a mean TM-score of 0.59 ± 0.1, which is indicative of a very good structural alignment (Xu and Zhang, 2010). Family composition does not cover all possible P-loop and Rossmann families: 13/26 and 4/13 families in the c.37.1 and c.2.1 superfamilies, respectively, are not involved in these hits. Supplementary Figure S3 depicts the number of hits between each family pair: leaving apart hits between the automated matched families (c.37.1.0 and c.2.1.0), the majority of hits involve the c.37.1.10, c.37.1.1 or c.2.1.2 families.

The hits between P-loop and Rossmann domains reveal that these two folds have homologous fragments, possibly at different regions of their sequences. To learn how many different types of fragments there are, about their lengths, and specific location in the proteins’ sequences, we used similarity networks.

### 3.2 View of evolutionary relationships via networks

**Fig. 4.**
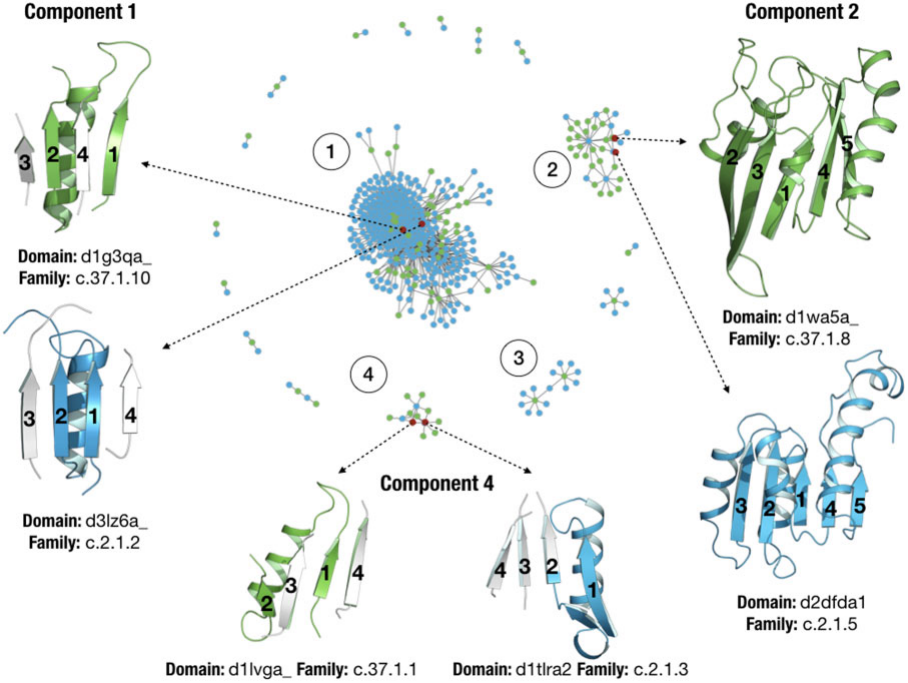
Similarity network for hits between the P-loop and Rossmann folds that surpass the thresholds (see main text). Nodes coloured in green and blue represent P-loop and Rossmann domains, respectively. Representative domains and their common fragments are shown for components 1, 2 and 4

Similarity networks allow the study and visualization of the protein universe or subregions of it, where the nodes represent protein domains and the links connect two domains when they have a fragment in common. In this case, we fetched 1737 hits that overall contain 432 different domains. The similarity network for these hits is shown in Figure 4. Domains belonging to the P-loop and Rossmann folds are coloured in green and blue, respectively. The network contains 460 nodes and 1213 links. For further details on the construction of the network and definitions we refer to our previous publication on Fuzzle (Ferruz *et al.*, 2020). Each of the ‘island-like’ motifs (called *components* in network theory) correspond to a set of domains that contain a common fragment. The network consists of 17 components with very diverse sizes. The largest component is composed of 361 nodes, whereas 7 components present only 2 nodes. We focus on three of the most populated components (Table 1).

**Table 1.** Summary of properties for components 1, 2 and 4 in the similarity network

Component	No. nodes (P-loop/Rossmann)	Size (aa.)	Fragment topology	Involved families (Excluding automated matches and singletons)
1	361 (48, 313)	33.7 ± 3.5	β1α1β2	c.2.1.2: 78; c.2.1.5: 20; c.2.1.6: 12; c.2.1.3: 4; c.2.1.7: 3; c.2.1.9: 2, c.2.1.4: 2; c.37.1.10: 14; c.37.1.1: 4; c.37.1.4: 2
2	35 (25, 10)	102.8 ± 2.1	β1β5α1α 4	c.2.1.5: 4; c.37.1.8: 20
4	13 (10, 3)	29.5 ± 1.6	β1α1β2	c.2.1.3: 3; c.37.1.1: 7

With 361 nodes component 1 is the major component of the network. The populations somewhat deviate towards Rossmann domains, with 48 and 313 nodes for the P-loop and Rossmann folds, respectively. These numbers can be appreciated in the component topology, with most Rossmann nodes connecting around P-loop nodes that act as *hubs*. Not surprisingly, the 16 most connected nodes in the component correspond to P-loop domains. Interestingly, all these nodes belong to either the c.37.1.10 family (Nitrogenase iron protein-like) or the c.37.1.0 family (automated matches) (Supplementary Fig. S4). On the other hand, most of the Rossmann domains belong to the c.2.1.2 family (tyrosine-dependent oxidoreductases). The nodes in this component share a fragment with an average length of 33.7 ± 3.5 amino acids which is located at the N-terminal end in both folds and consists of the β1α1β2 fragment, which contains both the Walker and Gly-rich motif. Figure 4 shows two representative P-loop and Rossmann domains for this component: d1g3qa_, the cell division regulator MinD from the c.37.1.10 family (Hayashi *et al*., 2001) and d3lz6a, 11-beta-hydroxysteroid dehydrogenase 1 protein from the c.2.1.2 family (Cheng *et al*., 2010). Representative domains shown in Figure 4 for each component are also depicted in red in the network.

The second largest component contains 35 nodes, with 25 and 10 P-loop and Rossmann domains, respectively. The fragment these domains have in common has an average length of 102.8 ± 2.2 amino acids and is defined by the first five strands and four helices (Fig. 4). The most connected domain is d2dfda1, a malate dehydrogenase NAD binding Rossmann domain, with 18 connections. Figure 4 shows two representative domains, d1wa5a_, a G-protein with an antiparallel β2, and d2dfda itself. The domains in this component, besides the families of automated matches, belong to the families c.37.1.8 (G-proteins) and c.2.1.5 (LDH N-terminal domain-like).

Component 3 contains 15 nodes, with two P-loop domains that connect to 13 Rossmann nodes, each connecting 7 domains. The Rossmann domain d2yv1a1, a succinyl-CoA binding domain, acts as a bridge between the two P-loop domains (Fig. 4). They correspond in fact to the same domain d2g0ta1, that due to slight differences in the sequence where the fragments are present was assigned to different clusters (d2g0ta1_2 and d2g0ta1_13). This domain belongs also to the c.37.1.10 family, but when looking in detail at its structure, we observe that it contains an extra N-terminal Rossmann fold which has not been classified by SCOPe as a different domain. The hit with the Rossmann domain d2yv1a1 is thus expected as both domains have the same fold. Component 3 exemplifies a case of misclassification occurring sometimes in hierarchical databases and as such it is not presented in detail in Table 1 and Figure 4.

Lastly, component 4 contains 13 nodes, with 3 Rossmann domains connecting to 10 P-loop domains. The most connected domain is d1t1ra2, a 1-deoxy-d-xylulose-5-phosphate reductoisomerase belonging to family c.2.1.3, with 7 connections (Yajima *et al*., 2004). The fragment in this component has an average length of 29.5 ± 1.6 amino acids. It also corresponds to the N-terminal βaβ-fragment containing the conserved motifs. Major differences with component 1 are the slightly smaller size of this fragment and the different families which contain it. While component 1 mainly contains c.37.1.10 domains, component 4 contains domains of the nucleotide and nucleoside kinase family (c.37.1.1). In this case, the network has discerned fragments into different components that are present in different P-loop families. Figure 4 depicts this fragment in two representative domains, d1lvga_ (Sekulic *et al*., 2002), a guanylate kinase from the nucleosides kinase family (c.37.1.1) and d1t1ra2 from family c.2.1.3.

### 3.3 Automatic construction of chimeras

Protlego builds all possible chimeras between two domains based on their sequence alignment (Section 2.3 and Fig. 2). Here we have created all possible chimeras between all P-loop/Rossmann pairs of domains (Supplementary Listing S1). As we previously noticed a misclassification of domain d2g0ta1 with an extra N-terminal Rossmann domain, we removed all its hits, leading to 1693 hits (previously 1737). We first performed chimeragenesis using a global alignment (Section 2.3), which led to a total of 1158 chimeras. Remarkably, only 5% of hits led to chimeras without backbone clashes, with hits presenting short alignment lengths producing very few chimeras (Supplementary Fig. S5a). Besides the average short length of these hits and their subsequent lower number of possible fusion points, another reason for the low number of chimeras is the intrinsic topology of the β-strands in the parents. The shifting of β2 in the strand order (23145 versus 213456) leads to backbone clashes once the two termini are combined. We decided to perform partial alignments instead, to see if stepwise alignments of shorter motifs improved the statistics. Indeed, the partial alignment provided 2503 chimeras coming from 27% of the hits, with hits of shorter lengths producing more chimeras (Supplementary Fig. S5b). Constructing chimeras for the 1693 hits took around 3 h on a desktop workstation (Supplementary Listing S1).

We had a look at those combinations of families that produced most chimeras. The combination of c.37.1.8 and c.2.1.5 led to 709 chimeras, despite only having 34 hits (Supplementary Fig. S6). In second place, the pair c.37.1.1 and c.2.1.3, gave 17 hits and produced a total of 104 chimeras. The third most abundant pair corresponds to families c.37.1.10 and c.2.1.2 that with 202 hits gave rise to 48 chimeras. Remarkably, these three examples exactly correspond to the predominant families found in component 2, 4 and 1, respectively (Fig. 4).

We focused on the first example. We built all possible chimeras between domains d1wa5a_ and d2dfda1, already chosen as representatives of component 2 in Figure 4. The hit has an HHsearch probability of 81.7%, a fragment length of 101 amino acids that superimpose with an RMSD of 2.89 Å over 85 Cα atoms and a TM-score of 0.55. The alignment identity is 14%. The algorithm first maps all amino acids in the sequence alignment to their corresponding positions in the PDB (Fig. 2).

In this case, the 101 amino acids were successfully mapped to the structures. Then, the partial alignment divided the (βα)_1-4_β_5_ fragment into six sections and distances between Cα pairs were computed. From the 101 aligned positions, a maximum of 202 offspring chimeras could be expected in the ideal scenario when the structures align perfectly, and the resulting chimeras do not have backbone clashes. In this case, 32 of those 101 points had a distance between Cα atoms below the default cut-off of 1 Å. From the possible 64 chimeras altogether in combination1 and 2, 43 of the built chimeras did not pass the last quality filter due to backbone-backbone clashes. Overall, 21 chimeras passed all criteria and were successfully built. Supplementary Figure S7 summarizes the outcome of each fusion point. This chimera building process took 6 s (Supplementary Listing S1).

Supplementary Figure S8 represents the 21 chimeras coloured according to the fragments they inherited from their parents (d1wa5a_: P-loop, green, d2dfda1: Rossmann, blue). Chimeras in combination1, (with d2dfda1 at the N-terminus) have the topology 321456, whereas the topology for chimeras in combination2 is strand order 23145.

### 3.4 Energy evaluation

We energetically evaluated the 21 chimeras with the Amber forcefield with backbone flexibility enabled. Results are summarized in Supplementary Table S2. Chimera scoring could be useful in contexts when only a few chimeras can proceed to the experimental assessment and fast means to rank them are necessary. As expected, the chimeras tend to score better when allowing backbone rearrangements during minimization. When ranking the chimeras by their score per residue, we observe that the first four correspond to the chimeras in combination2 with the most P-loop content (comb2_109-118). In the middle part of the table, we find mostly chimeras from combination1 that contain three β-strands from each parent. The last part of the ranking is mostly populated with combination2 chimeras with less P-loop content (comb2_80-107). One possibility explaining the striking differences observed between chimeras from comb2_80-107 and comb2_109-118 is that the first group involves a fusion point right in the middle of the helix coming from β4 while the four chimeras in comb2_109-118 conserve the native helix from the P-loop domain (Supplementary Fig. S8). We have also minimized and scored the parent domains to allow direct comparison. Interestingly, the parent domains have scores at the two extremes of the chimera distribution: domain d1wa5a_, confers the better scoring domain (-22.1 kcal/mol) and domain d2dfda1 the worst (-18.1 kcal/mol). We questioned whether the order in Supplementary Table S2 reflects the partial content of the two chimeras, with higher-scoring chimeras having more P-loop content. Although some trend is observed for the first chimeras, with high P-loop content and very negative scores, an overall trend between the two variables could not be found (*R*^2^ = 0.25).

### 3.5 Structural analysis

Protlego enables the analysis of several structural features, such as hydrophobic clusters, hydrogen bond plots (Bikadi *et al.*, 2007), salt-bridge and hydrogen-bond networks, solvent-accessible surface area (SASA), contact orders and contact map representations, among others. Here, we show some of these analyses with chimera comb1_72, as it provides an interesting structure with three inherited β-strands from each parent and being one of the highest scoring members in the set.

**Fig. 5.**
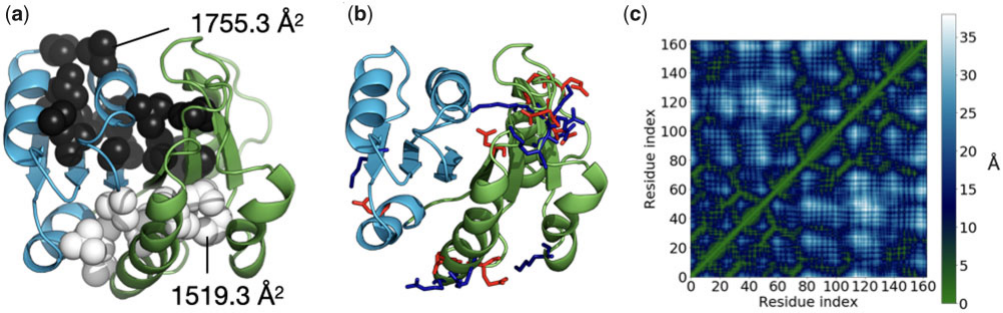
Some of the structural analysis included in Protlego. (**a**) The two largest hydrophobic clusters shown in black and in white computed for chimera comb1_72. (**b**) Computed salt bridges. Acidic residues are shown in red and basic in blue. (**c**) Computation of contact maps. Distances color-coded and listed in Å: Regions in green are close whereas those in blue are far apart

We started by analyzing hydrophobic clusters (Section 2.5 and Supplementary Text S1). To allow direct comparison, we performed the same computation on the parents d1wa5a_ and d2dfda1, in all cases after minimization. Both parents contain two main hydrophobic clusters, flanking both sides of the β-sheet (Supplementary Fig. S9). While clusters in domain d2dfda consist of 16 (black) and 15 (white) residues, both clusters in domain d1wa5a_ are composed of 9 residues, despite d1wa5a_ containing more residues overall (172 versus 147). We have often observed that the chimeras do not inherit the hydrophobic cluster conformation from their parent proteins, due to non-hydrophobic new residues in places that can break the cluster continuity. However, comb1_72 contains two major clusters consisting of 16 and 11 residues (Fig. 5c and Supplementary Fig. S9c). Although the largest cluster fails to reproduce the large area of the Rossmann parent d2dfda1 (1996.7 versus 1755.3 Å^2^) it contains residues from the parent P-loop domain d1wa5a_, forming a continuous entity. A similar behaviour is observed for the second cluster which covers an area of 1519.3 Å^2^ with 11 residues spanning the two regions.

We then turned to the computation of salt bridges. The Rossmann domain contains fewer salt bridges, summing up to only five (Supplementary Fig. S10a). The P-loop domain, however, has a total of 14 salt bridges, mostly located in the region defined by the last three β-strands (Supplementary Fig. S10b). Comb1_72 contains 9 salt bridges, 8 of them inherited from parent d1wa5a_ (Supplementary Fig. S10c). Comb1_72 forms a new interaction between β3 and α3, perhaps a consequence of repacking between α3 and α4 after minimization. The salt bridge between β1 and β4 on the other hand was lost after fusion.

On a different note, contact maps reduce the dimensionality of proteins to a 2D-plot that reveals which positions in the protein are close in space. An advantage of contact maps is that they are invariant to rotations and translations and often used for protein superimposition (Holm and Sander, 1996). As expected, the chimera contact map presents a combination of the maps of their parents, with residues 1–71 reflecting the Rossman map, and 71–165 resembling the P-loop map (Fig. 5 and Supplementary Fig. S11).

Another interesting property is the contact order, which describes the average sequence distance between residues that are in structural contact. Contact orders have been studied in the context of protein folding, and it is suggested that higher contact orders indicate longer folding times (Plaxco *et al*., 1998). Interestingly, designed proteins tend to have lower contact orders than distributions observed in natural proteins (Bonneau *et al*., 2002). Their typical value ranges anywhere from 5% to 25%. In line with these, parent domains and chimera have a contact order of 14.1% (d2dfda1), 18.3% (d1wa5a_), and 15.2% (comb1_72), respectively.

Solvent-accessible surface area is the area of a protein that is accessible to a solvent molecule. For chimera comb1_72 we calculate a total area of 8470 Å^2^, the Rossmann domain d2dfda1 has a total area of 7340 Å^2^ and the P-loop domain d1wa5a_ contains surfaces of 8870 Å^2^ in total.

## 4 Conclusion

The design of novel proteins via recombination of sub-domain sized fragments provides an attractive new route for the design of customizable proteins. The modelling and ranking of several hundreds of chimeric proteins prior to its testing in the lab requires however the use of several techniques and a considerable degree of expertise. Here, we have implemented Protlego, a Python-based open-source software for the automatic construction of chimeras and their structural analysis. Protlego contains a lightweight version of the Fuzzle database which enables fetching hits from specific SCOPe groups or that fulfil a specific user-defined criterion. The retrieved Fuzzle hits can then be represented via similarity networks, facilitating the understanding of convoluted evolutionary relationships. Selected hits can be used to build chimeras with one recombination point, whose structural features can be further analyzed in detail, or their potential energies estimated and ranked.

In this work, we chose to showcase Protlego’s features on a biologically relevant example that has attracted the attention of many groups in the past: The relationship of the P-loop and Rossmann folds (Longo* et al.*, 2020) . By fetching hits from Fuzzle, we obtained a total of 1737 domains of these two folds. We represented these hits via a network, which nicely separated their different fragments by length, position and family relationships. In fact, not all P-loop and Rossmann families are homologous, rather only a subset of them, with families c.37.1.10 and c.2.1.2 being predominantly connected (Fig. 4). We selected the hit between domains d1wa5a_ and d2dfda1 to illustrate the process of automatic chimera construction. In particular, this hit leads to 21 chimeras with different parent content and estimated potential energies (Supplementary Table S2). We selected one chimera for further analysis, which indicated that it is potentially well-folded. Specifically, the hydrophobic clusters coming from the two parents coalesced nicely spanning the two combined regions, and other features showed values similar to those of natural proteins.

Despite these observations, the correlation between structural features and experimental success is not yet clear. Protlego provides a comprehensive set of analysis tools, such as potential energy calculations and easy-to-use characterization of the most commonly studied intramolecular interactions. These can be used to guide the selection of a few designs for experimental validation. In the long run, once more chimeras have been tested and biochemical and structural data of a broader set are available, this will in turn provide data for future benchmarking and further development of chimera scoring. We made Protlego available on conda and GitHub at https://github.com/Hoecker-Lab/protlego and welcome participation from the scientific community. Examples, documentation and installation guidelines are also available.

## Supplementary Material

btab253_Supplementary_Data
