# Supplementary material for: Protlego: a Python package for the analysis and design of chimeric proteins
Source: Bioinformatics. 2021 Apr 26;37(19):3182–9. doi: 10.1093/bioinformatics/btab253 (PMC8504633; doi:10.1093/bioinformatics/btab253)
Supplement: btab253_Supplementary_Data [file btab253_supplementary_data.pdf]

Supporting Information for:

Protlego: A Python package for the analysis and design of chimeric proteins

Table of Contents

Listing S1: Code used to generate the results in the manuscript. 2

Text S1: Computation of hydrophobic clusters: a Python reimplementation of the CSU algorithm 3

Table S1: Fetching functions to retrieve from the built-in Fuzzle database 4

Table S2: Summary of the energy minimization with the Amber forcefield. 4

Figure S1: Summary of the algorithm to compute hydrophobic clusters. 5

Figure S2: Distribution of fragment length for fragments in the retrieved hits. 6

Figure S3: Number of hits found between each P-loop and Rossmann family. 7

Figure S4: Similarity network colored by families. 8

Figure S5: Relationship of alignment length and number of produced chimeras for the global (a) and partial (b) alignments. The chimeras were built taking into account all hits. 8

Figure S6: Combination of families and the number of chimeras they produced. 9

Figure S7: Summary of chimera outcomes for each alignment position. 9

Figure S8: Representation of all chimeras produced for the selected hit. 10

Figure S9: The two largest hydrophobic clusters found in the parent domains and chimera comb1_72. 10

Figure S10: Salt bridges for parent domains and chimera. 11

Figure S11: Contact maps for parent domains and chimera. 11

LISTINGS

Listing S1: Code used to generate the results in the manuscript. Computational costs for each section are including as comments.

```
# Importing the module
from protlego.all import *

# Retrieving all hits between the two folds # time: 8.39 seconds
hits=fetch_group('c.37','c.2')

# Creating network and plotting # time: 2.82 seconds
a=Network(hits)
graph = a.create_network()
a.plot_graph(graph,'fold')

# Building all possible chimeras. # time: 12,807 s
chimeras={}
for index,hit in enumerate(hits.hits):
    a=Builder(hit)
    aln=a.get_alignment(hit.query,hit.no)
    a.superimpose_structures(aln,partial_alignment=True)
    chimeras[index]=a.build_chimeras(partial_alignment=True)

# Selecting one hit and scoring its chimeras # time: 6.38 s
selected_hit=hits[382]
b=Builder(selected_hit)
aln=a.get_alignment(selected_hit.query, selected_hit.no)
b.superimpose_structures(aln,partial_alignment=True)
sel_chimeras=b.build_chimeras(partial_alignment=True)
b.plot_curves(selected_hit.query)

# Minimizing all chimeras # time: 837 s
values_amber={}
chimeras_after_amber={}
for key,chimera in sel_ chimeras.items():
    values_amber[key],chimeras_after_amber[key]=minimize_potential_energy(chimera,
'amber', restraint_backbone=False)

# Structural analysis of chimera comb1_72 # Time: 199.1 s
chimera = chimeras_after_amber['comb1_72']
clusters = chimera.compute_hydrophobic_clusters()
chimera.view()
chimera_salts = chimera.compute_salt_bridges()
chimera.view()
calc_sasa(chimera)
calc_contact_order(chimera)
distance_matrix=calc_dist_matrix(chimera,type='distances', plot=True)
hnets=chimera.compute_hydrogen_networks()
calc_contact_order(chimera)
chimera.view()
```

TEXT

Text S1: Computation of hydrophobic clusters: a Python reimplementation of the CSU algorithm.

It has been proposed that sidechains of isoleucine (ILE), leucine (LEU) and valine (VAL) often form hydrophobic or so-called (ILV)- cluster that prevent the intrusion of water molecules and serve as cores of stability in high-energy partially folded states [1]. Although still not well understood, hydrophobic clusters seem to play a key role in protein stability [2]. An available source to compute hydrophobic clusters is the BASIC web server, which relies on the CSU algorithm [3–5]. The CSU algorithm was released as a web server application but is unfortunately no longer maintained. We thus implemented the original CSU algorithm in Protlego to enable its use in a high-throughput fashion. The `compute_hydrophobic_clusters` function allows computing cluster for user-defined selections and visualizing them in the protein structure. **Fig. S1** summarizes the algorithm, which proceeds as follows: Two atoms A and B are considered to be in contact if a solvent molecule placed at the surface of A's sphere, overlaps with the sphere formed by a solvent molecule plus the Van der Waals sphere of atom B [6]. The atoms are considered spheres of fixed radius, obtained from a previous publication [7]. If at any position a water molecule penetrates several atoms' spheres, the contact is considered to belong to that whose centre is closest to the centre of atom A. In practical terms, Protlego defines an `Atom` class for each heavy atom that fulfils the user selection (such as residues ILE, VAL, and LEU). During instantiation, the `Atom` class retrieves the coordinates of the neighbouring atoms. These are atoms that are closer than the sum of the two Van der Waals radii, each enlarged by the radius of the water molecule (1.4). Hence, for two carbon atoms to be considered candidates for atomic contacts they must be within 6.56 Å. The `Atom` class then discretizes the sphere of the atom in question into many uniform small sections. We use the Fibonacci grid [5,8] to perform the discretization and select 610 points by default. The area corresponds to 0.0016 of the total area of the sphere. The algorithm then evaluates if any of the 610 (or a user-defined number) sections overlaps with the neighbours, and if so, the contact in the section is declared to belong to the sphere whose centre is closest to A's centre.

The algorithm is followed for all the atoms until a matrix of residue-against-residue areas is computed. By default, we define that two residues are in contact when they have an overlapping area of at least 10 Å^2. The adjacent matrix is converted to a graph, where every component corresponds to a (hydrophobic) cluster. The total area of the cluster is computed by the sum of the individual residue areas that comprise it.

TABLES

Table S1: Fetching functions to retrieve from the built-in Fuzzle database.

Fetching function	Hits that the function fetches from Fuzzle
`fetch_id`	Hit with that Fuzzle ID
`fetch_by_domain`	Hits that contain a specific domain as query or subject
`fetch_by_domains`	Hits between the two domains
`fetch_by_PDB`	Hits that contains domains that belong to the PDB
`fetch_by_PDBs`	Hits between two domains that belong to PDB1 and PDB2
`fetch_group`	Hits belonging to one or hits between two specific SCOPe groups (folds, superfamilies, families).
`Fetch_subspace`	Hits that satisfy the cut-offs for RMSD, length, and TM-score, among others.

All include RMSD, minimum and maximum lengths, or TM-score as optional parameters to refine the search.

Table S2: Summary of the energy minimization with the Amber forcefield. The minimizations were performed allowing for backbone relax or not. The table is ordered according to the best scoring chimeras without backbone restraints.

Chimera or domain Name	Score per residue (kcal/mol)	P-loop content (%)
**d1wa5a_**	-22.1	100
**comb2_118**	-21.9	78.9
**comb2_112**	-21.3	74.6
**comb2_113**	-21.3	75.3
**comb2_109**	-21.0	72.5
**comb1_72**	-20.3	58.9
**comb2_75**	-20.2	50.0
**comb1_79**	-20.2	54.6
**comb1_77**	-20.1	55.8
**comb1_78**	-20.1	55.2
**comb1_81**	-20.1	53.4
**comb1_76**	-19.9	50.7
**comb2_76**	-19.9	56.4
**comb2_103**	-19.7	68.3
**comb2_105**	-19.7	69.7
**comb1_73**	-19.6	58.3
**comb2_80**	-19.6	53.3
**comb2_106**	-19.6	70.4
**comb2_81**	-19.5	53.9
**comb2_104**	-19.5	69.0
**comb2_107**	-19.4	71.1
**comb1_74**	-19.3	57.7
**d2dfda1**	-18.2	0

FIGURES

Figure S1: Summary of the algorithm to compute hydrophobic clusters. Given a PDB, an Atom class is defined for each heavy atom in the given selection (by default ILE, VAL and LEU residues) (**1**). The atom is represented as a sphere whose surface is divided into 610 sections (or a user defined number). Each section is evaluated whether it intersects another Atom object or not (**2**). The total areas are summed up per residue and a matrix is built (**3**). The matrix can be transformed into a graph (**4**), whose components virtually correspond to the fragments in the protein (**5**)

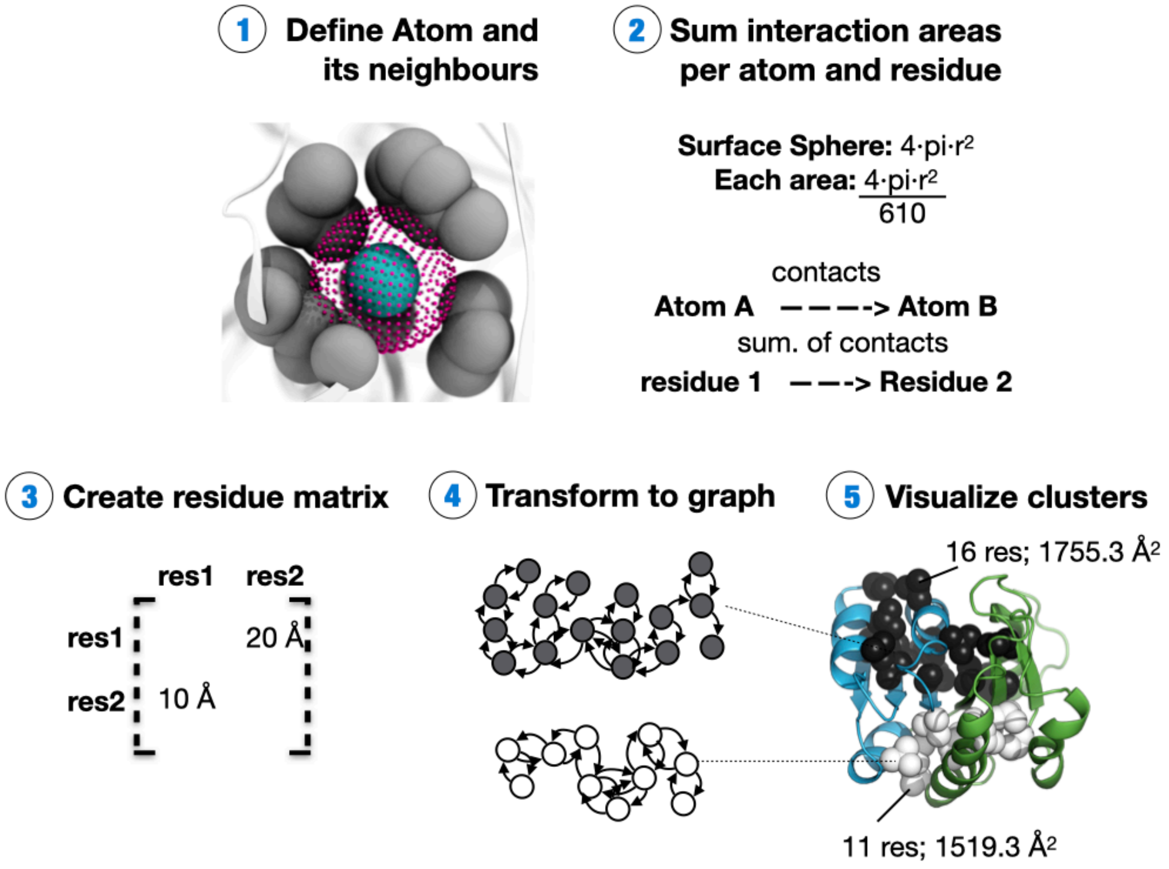

Figure S2: Distribution of fragment length for fragments in the retrieved hits. In blue, the histogram including all hits (1737), in orange, excluding domain d2g0ta1 due to misclassification (1693). The two sets have virtually identical distributions.

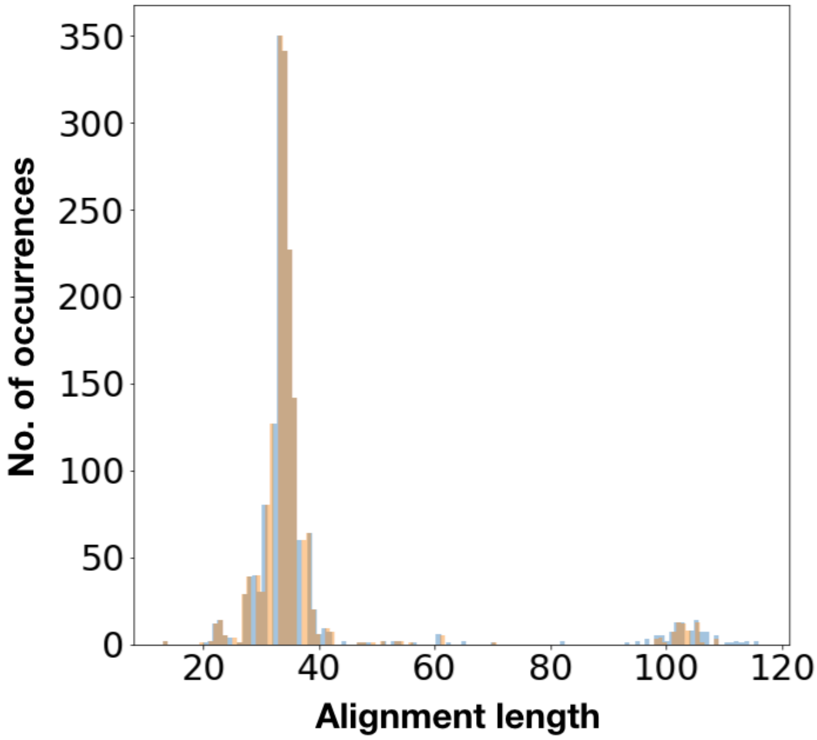

Figure S3: Number of hits found between each P-loop and Rossmann family. Besides the hits between automated matches (c.37.1.0 and c.2.1.0), the majority of hits involve the c.37.1.10 or c.2.1.2 families. Column and row 1 are depicted in gray as they represent the families of automated matches. Column 10' indicates the number of chimeras produced by family c.37.1.10 when removing hits that include domain d2g0ta1 as query or subject due to misclassification (see main manuscript). Greater numbers are depicted in darker shades of green.

P-loop families 'c.37.1.x' (columns); Rossmann families 'c.2.1.x' (rows)

	0	1	2	3	4	5	6	7	8	9	10	10'	11	12	14	15	16	17	18	19	20	21	22	23	24	25	26
0	361	31		2	6	2			23		552	550								5						1	
1	1										1	1															
2	177	14		4	14						202	202				4											
3	17	17									25	2											2				
4	7										10	10															
5	33	7							34		37	37															
6	37										51	51															
7	6								1		5	5			2												
8											19	0															
9	10	2				1					14	14															
11												0															
12												0															
13												0															

Figure S4: Similarity network colored by families. Each family in the network is automatically assigned a color by Protlego. The different components in the network have different family contents.

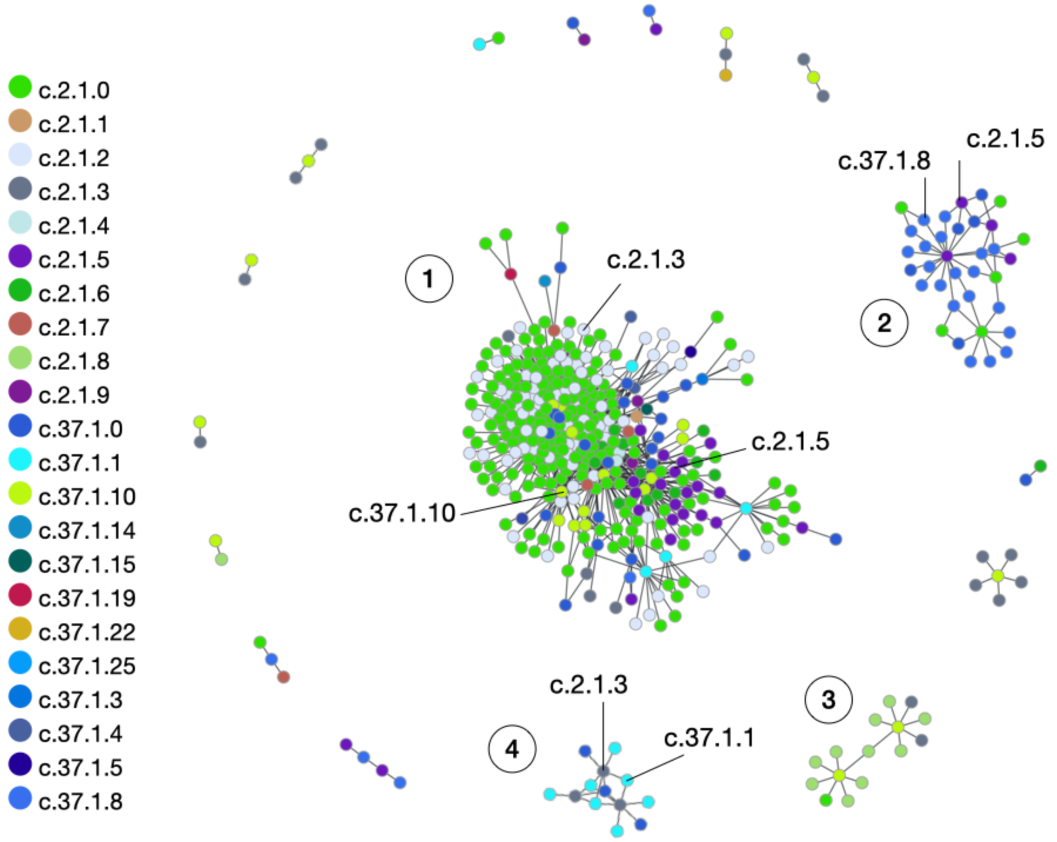

Figure S5: Relationship of alignment length and number of produced chimeras for the global (a) and partial (b) alignments. The chimeras were built taking into account all 1737 hits between P-loop and Rossmann domains.

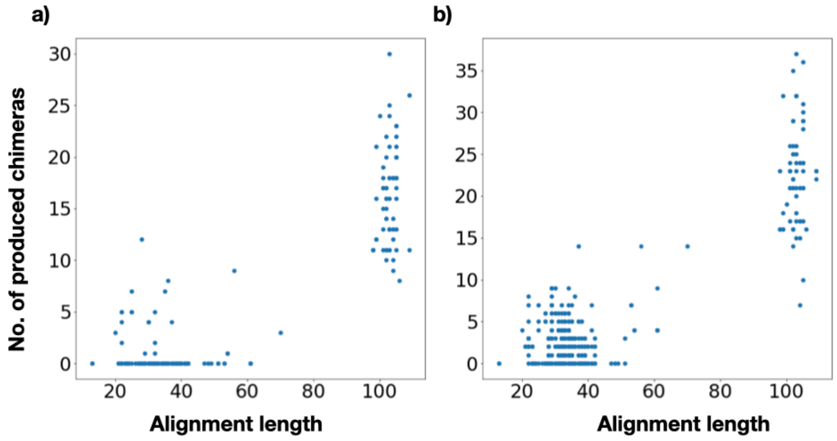

Figure S6: Combination of families and the number of chimeras they produced. The numbers correspond to the partial alignment algorithm. The total number of chimeras is 3170 when counting all domains, and 2503 when removing domain d2g0ta1. Column and row 1 are depicted in gray as they represent the families of automated matches. Column 10' indicates the number of chimeras produced by family c.37.1.10 when removing hits containing d2g0ta1 due to misclassification. The Figure is represented such as **Fig. S3**, with larger numbers being depicted in darker shades of green.

P-loop families 'c.37.1.x' (columns); Rossmann families 'c.2.1.x' (rows)

	0	1	2	3	4	5	6	7	8	9	10	10'	11	12	14	15	16	17	18	19	20	21	22	23	24	25	26
**0**	329	71		9					508		206	188								25							
**1**	7										4	4															
**2**	112	16		11	4						48	48															
**3**	54	104									152	0											8				
**4**											9	9															
**5**	174	2							709		32	32															
**6**	35											0															
**7**	3										2	2			14												
**8**											497	0															
**9**		4									3	3															
**11**												0															
**12**												0															
**13**												0															

Figure S7: Summary of chimera outcomes for each alignment position. Out of the 101 alignment positions, 31 present distances between Cα pairs below 1 Å. 24 and 19 of these points produce a chimera with clashes (shown in black). 21 final chimeras are buildable for combination 1 (yellow) and 2 (red)

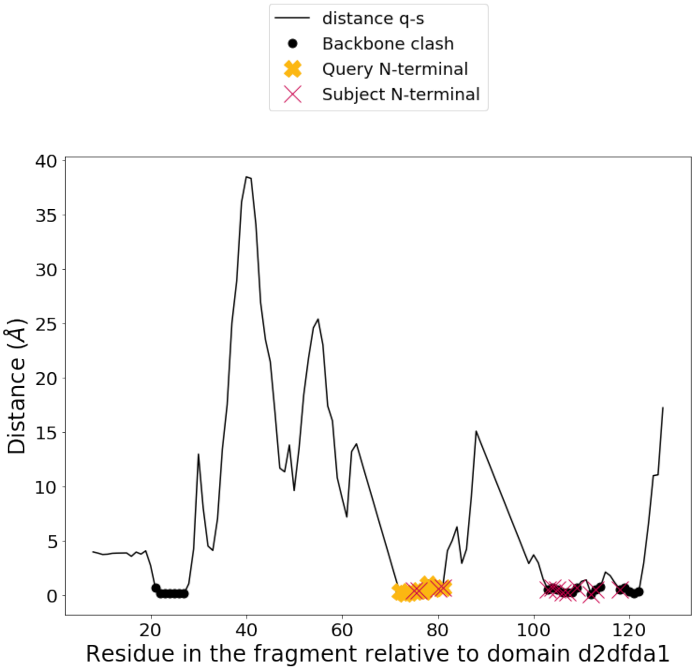

Figure S8: Representation of all chimeras produced for the selected hit. The hit between the domains d2dfda1 (query, Rossman) and d1wa5a_ (subject, P-loop) produced 21 offspring chimeras. Names for each chimera are depicted below its representation. The number summarizes the combination that it comes from (comb1 or comb2) and the residue where the parents are joined. Chimeras in combination 1 have a topology of strand order 321456, whereas chimeras in combination 2 the strand order 23145. The colouring method preserves the previous representation for the parents (blue: Rossmann, green: P-loop)

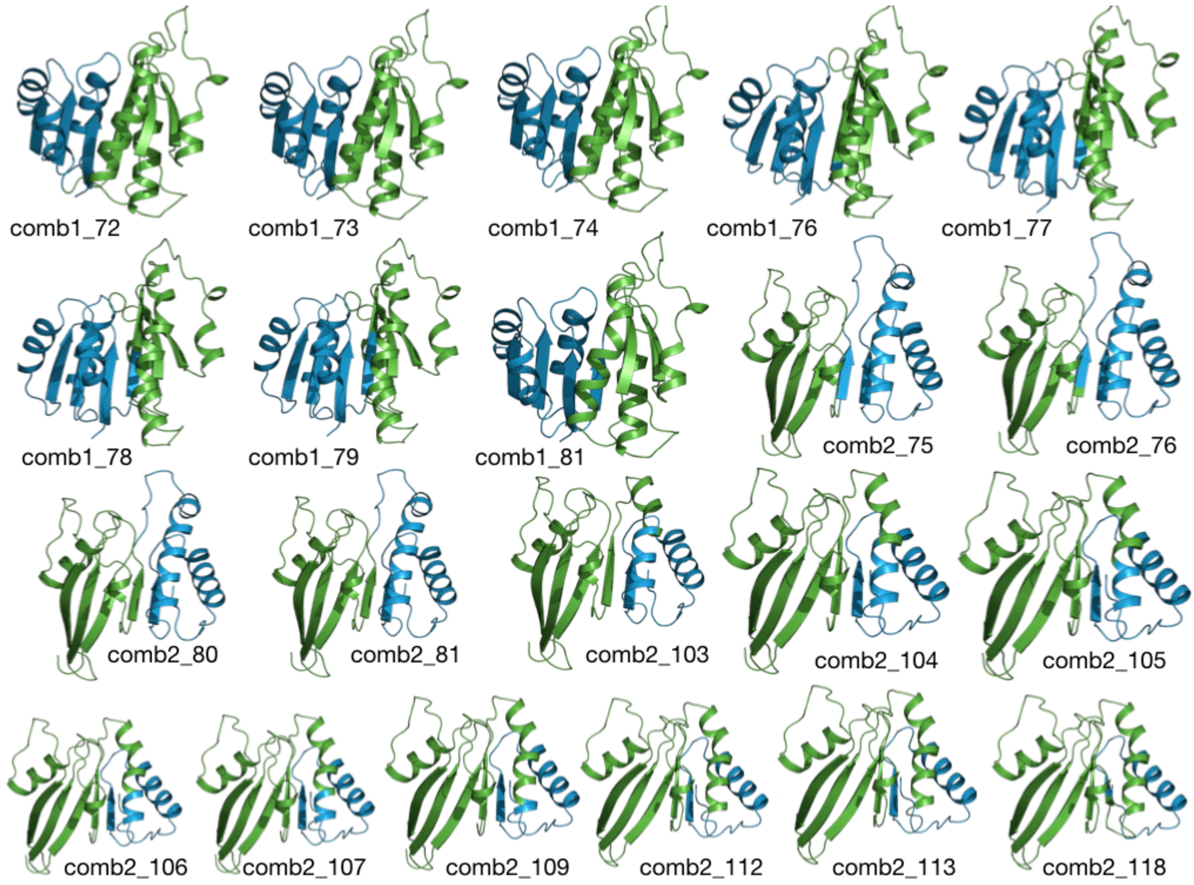

Figure S9: The two largest hydrophobic clusters found in the parent domains and chimera comb1_72. Largest and second largest clusters are depicted in black and white, respectively.

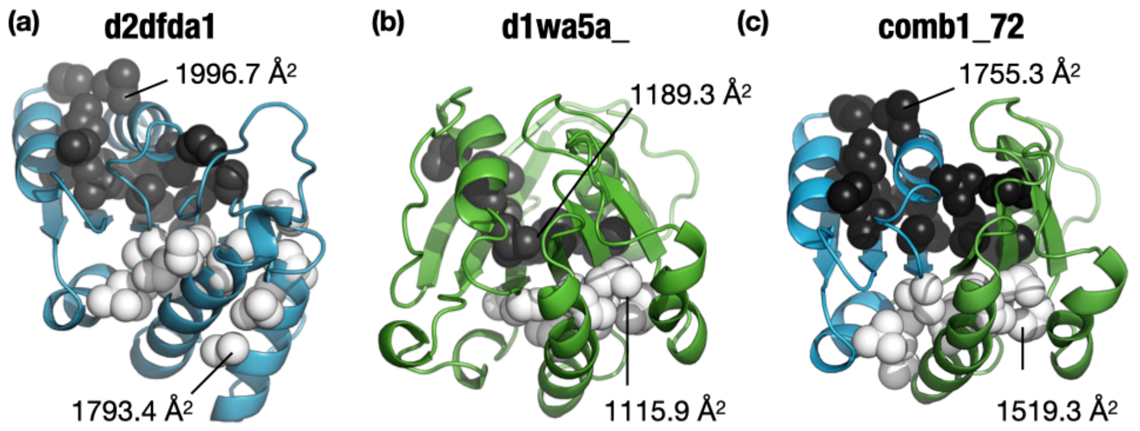

Figure S10: Salt bridges for parent domains and chimera. Acidic and basic residues are shown in red and blue, respectively.

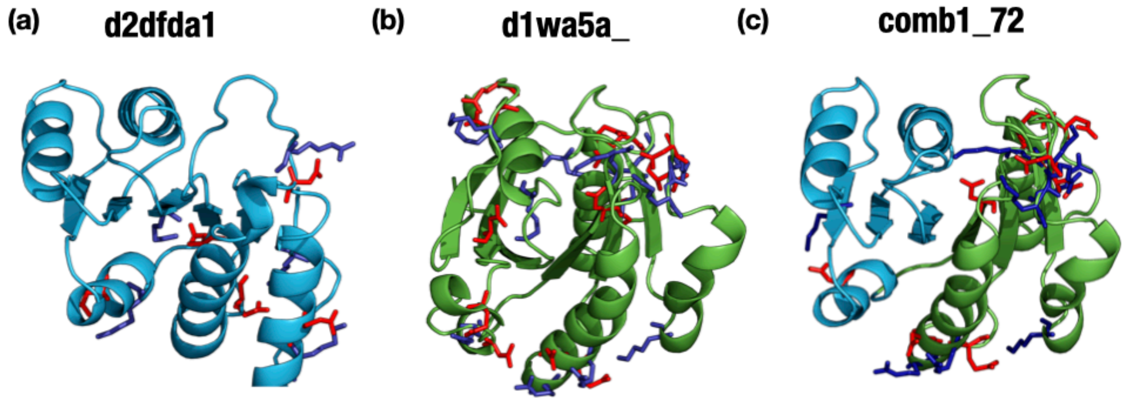

Figure S11: Contact maps for parent domains and chimera. Residues close in distance are shown in green, whereas those far apart are shown in different shades of blue. Other color representations are possible.

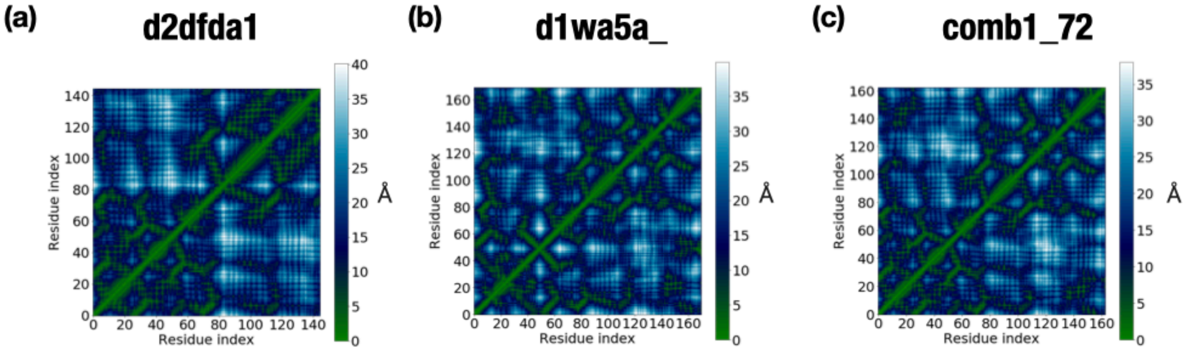

SUPPLEMENTARY REFERENCES

1. Kathuria, S. V *et al.* Clusters of isoleucine, leucine, and valine side chains define cores of stability in high-energy states of globular proteins: Sequence determinants of structure and stability. *PROTEIN Sci.* **25**, 662–675 (2016).
2. Basak, S. *et al.* Networks of electrostatic and hydrophobic interactions modulate the complex folding free energy surface of a designed βα protein. *Proc. Natl. Acad. Sci. U. S. A.* **116**, 6806–6811 (2019).
3. Sobolev, V., Sorokine, A., Prilusky, J., Abola, E. E. & Edelman, M. Automated analysis of interatomic contacts in proteins. *Bioinformatics* **15**, 327–332 (1999).
4. Sobolev, V., Wade, R. C., Vriend, G. & Edelman, M. Molecular docking using surface complementarity. *Proteins Struct. Funct. Bioinforma.* **25**, 120–129 (1996).
5. Wołek, K., Gómez-Sicilia, À. & Cieplak, M. Determination of contact maps in proteins: A combination of structural and chemical approaches. *J. Chem. Phys.* **143**, (2015).
6. Sobolev, V. & Edelman, M. Modeling the quinone-B binding site of the photosystem-II reaction center using notions of complementarity and contact-surface between atoms. *Proteins Struct. Funct. Bioinforma.* **21**, 214–225 (1995).
7. Shannon, R. D. Revised effective ionic radii and systematic studies of interatomic distances in halides and chalcogenides. *Acta Crystallogr. Sect. A* **32**, 751–767 (1976).
8. González, Á. Measurement of Areas on a Sphere Using Fibonacci and Latitude-Longitude Lattices. *Math. Geosci.* **42**, 49–64 (2010).
